# TRAIL attenuates RANKL-mediated osteoblastic signalling in vascular cell mono-culture and co-culture models

**DOI:** 10.1371/journal.pone.0188192

**Published:** 2017-11-16

**Authors:** Emma Harper, Keith D. Rochfort, Hannah Forde, Colin Davenport, Diarmuid Smith, Philip M. Cummins

**Affiliations:** 1 School of Biotechnology, Dublin City University, Dublin, Ireland; 2 National Institute for Cellular Biotechnology, Dublin City University, Dublin, Ireland; 3 Department of Academic Endocrinology, Beaumont Hospital, Dublin, Ireland; Centro Cardiologico Monzino, ITALY

## Abstract

**Background and objectives:**

Vascular calcification (VC) is a major risk factor for elevated cardiovascular morbidity/mortality. Underlying this process is osteoblastic signalling within the vessel wall involving complex and interlinked roles for receptor-activator of nuclear factor-κB ligand (RANKL), osteoprotegerin (OPG), and tumour necrosis factor-related apoptosis-inducing ligand (TRAIL). RANKL promotes vascular cell osteoblastic differentiation, whilst OPG acts as a neutralizing decoy receptor for RANKL (and TRAIL). With respect to TRAIL, much recent evidence points to a vasoprotective role for this ligand, albeit via unknown mechanisms. In order to shed more light on TRAILs vasoprotective role therefore, we employed *in vitro* cell models to test the hypothesis that TRAIL can counteract the RANKL-mediated signalling that occurs between the vascular cells that comprise the vessel wall.

**Methods and results:**

Human aortic endothelial and smooth muscle cell mono-cultures (HAECs, HASMCs) were treated with RANKL (0–25 ng/mL ± 5 ng/mL TRAIL) for 72 hr. Furthermore, to better recapitulate the paracrine signalling that exists between endothelial and smooth muscle cells within the vessel wall, non-contact transwell HAEC:HASMC co-cultures were also employed and involved RANKL treatment of HAECs (±TRAIL), subsequently followed by analysis of pro-calcific markers in the underlying subluminal HASMCs. RANKL elicited robust osteoblastic signalling across both mono- and co-culture models (e.g. increased BMP-2, alkaline phosphatase/ALP, Runx2, and Sox9, in conjunction with decreased OPG). Importantly, several RANKL actions (e.g. increased BMP-2 release from mono-cultured HAECs or increased ALP/Sox9 levels in co-cultured HASMCs) could be strongly blocked by co-incubation with TRAIL. In summary, this paper clearly demonstrates that RANKL can elicit pro-osteoblastic signalling in HAECs and HASMCs both directly and across paracrine signalling axes. Moreover, within these contexts we present clear evidence that TRAIL can block several key signalling actions of RANKL in vascular cells, providing further evidence of its vasoprotective potential.

## Introduction

Vascular calcification (VC) afflicts multiple patient populations, where it manifests as elevated levels of mineral deposition within the intimal and medial regions of blood vessels. This has adverse consequences for vessel wall homeostasis, and constitutes a significant risk factor for elevated rates of cardiovascular (CV) morbidity and mortality [1,2]. VC can inhibit cardiac valve function, decrease arterial compliance, and increase the risk of post-angioplasty dissection [3]. Moreover, VC is associated with increased risk of plaque rupture, aortic stiffness, left ventricular hypertrophy, and increased pulse pressure, and is also significantly elevated in diabetic and chronic kidney disease sufferers, where it contributes to the premature ageing of blood vessels [4–6].

Driving the VC process, much evidence now points to coordinated roles for a signalling triad comprising receptor-activator of nuclear factor-κB ligand (RANKL), osteoprotegerin (OPG), and tumour necrosis factor-related apoptosis-inducing ligand (TRAIL) [7,8]. Within the vasculature, RANKL is known to exhibit pro-calcific actions, with RANKL expression distinctly elevated in areas of mineralization [9]. Consistent with this, numerous studies have demonstrated that RANKL may promote trans-differentiation of vascular smooth muscle cells (VSMCs) to an osteoblastic/chondroblastic phenotype either through direct interaction with the VSMC RANK receptor [10,11], or by inducing the release of endothelial-derived pro-osteoblastic paracrine signals such as bone morphogenetic protein-2/4 (BMP-2/4), which in turn may act upon the underlying VSMCs [12,13]. OPG, produced in substantial quantities by VSMCs (and non-vascular sources), acts as a soluble decoy receptor for RANKL to neutralize its biological actions within the vasculature [14]. This anti-calcific action of OPG within the vessel wall is supported by research demonstrating that OPG^-/-^ mice display severe VC burden [15], whilst atherogenic mice treated with recombinant OPG exhibit reduced levels of plaque calcification and aortic osteocalcin [16]. It is also noteworthy that the roles for RANKL and OPG within the vascular wall are, paradoxically, opposite to those observed for these same ligands within bone morphogenesis [8].

OPG can also serve as a decoy receptor for TRAIL [17], although the precise role of the latter ligand within the VC process is much less well understood. In this respect however, a growing body of *in vivo* and clinical evidence now points to a vasoprotective role for TRAIL (for review, see [18]). For example, circulating TRAIL levels are decreased following acute myocardial infarction and heart failure, whilst lower levels of TRAIL following an acute CV event are associated with increased mortality [19–21]. Serum TRAIL levels are also inversely correlated with carotid intimal-medial thickness [22]. In animal studies, diabetic mice have shown improvements in glucose clearance, insulin sensitivity, and normoglycaemic duration following recombinant TRAIL treatment [23], whilst exogenous TRAIL administration has also demonstrated anti-atherosclerotic activity in Apo-E^-/-^ diabetic mice [24]. Of more direct relevance to VC and the present study, a recent paper by di Bartolo and co-workers has demonstrated how TRAIL deficiency leads to accelerated VC in ApoE^-/-^ TRAIL^-/-^ mice [25]. Moreover, previous work by Zauli *et al*. has demonstrated the ability of TRAIL to counteract RANKL signalling in human peripheral blood osteoclastic precursors and Raw264.7 murine monocytic cells [26,27].

These latter observations by Zauli *et al*. [26,27] have therefore led us to hypothesize that TRAIL may elicit its vasoprotective effects in-part by attenuating RANKL-induced osteoblastic signalling in vascular cells. The specific goal of this paper was to address this important hypothesis using *in vitro* vascular cell models. We have employed HAECs and HASMCs to profile a broad array of RANKL effects, and to investigate the influence of TRAIL upon these effects. Mono-culture experiments were initially employed, followed by a more physiologically relevant non-contact co-culture model designed to better recapitulate the paracrine signalling properties of the vessel wall. For these studies, a wide range of signalling molecules were monitored, including OPG, BMP-2, alkaline phosphatase (ALP), runt-related transcription factor-2 (Runx2, an osteogenic transcription factor), and Sox9 (a chondrocytic transcription factor). Our findings not only broaden our understanding of how RANKL may potentially elicit calcific activation both within and between vascular cells, but they also clearly indicate for the first time that TRAIL can attenuate specific actions of RANKL.

## Materials and methods

Unless otherwise stated, all reagents were purchased from Sigma-Aldrich (Dublin, IRL). Both human aortic endothelial cells (HAECs) and human aortic smooth muscle cells (HASMCs), as well as their respective growth media were purchased from Promocell GmbH (Heidelberg, Germany). Recombinant human RANKL and TRAIL were purchased from R&D Systems (Minneapolis, MN, USA). Tumor necrosis factor-alpha (TNF-α) was purchased from Merck Millipore (Danvers, MA, USA). Primers were sourced from Sigma Aldrich and Eurofins Genomics (Ebersburg, Germany). ELISA DuoSet kits and alkaline phosphatase (ALP) activity assay kits were purchased from R&D Systems and BioAssay Systems (Hayward, CA, USA), respectively, whilst qPCR reagents were purchased from Applied Biosystems/ThermoFisher Scientific (Paisley, UK).

### Guidelines and ethical approval

All experimental protocols were carried out in accordance with Dublin City University health and safety regulations. Institutional ethical approval and informed consent were not required for this study (i.e. use of widely commercially available human-derived cell lines).

### Cell culture

HAECs obtained from a 23 year old Caucasian male were cultured in endothelial cell growth medium (Promocell GmbH, catalog no. C22020) with the following supplements; fetal calf serum (0.05 mL/mL), endothelial cell growth supplement (0.004 mL/mL), epidermal growth factor (10 ng/mL), heparin (90 μg/mL) and hydrocortisone (1 μg/mL). This media was also supplemented with penicillin (100 IU/mL) and streptomycin (100 μg/mL). HASMCs obtained from a 19 year old Caucasian male were cultured in smooth muscle cell growth medium (Promocell GmbH, catalog no. C22062) containing the same concentrations of antibiotics, in addition to fetal calf serum (0.05 mL/mL), epidermal growth factor (0.5 ng/mL), basic fibroblast growth factor (2 ng/mL), and insulin (5 μg/mL). Cells were maintained in a humidified incubator at 37°C and 5% CO_2_. Passages 5–10 were used for experimental purposes. Cell number and viability were routinely measured using the advanced detection and accurate measurement (ADAM^™^) cell counter (Digital Bio, Seoul, KOR), both for seeding density purposes and to allow normalization of results where necessary. This process involved the digital analysis of cells on AccuChip slides following the addition of propidium iodide with or without a membrane permeabilizing solution, providing total numbers of both viable and non-viable cells. Cell culture experiments were subsequently conducted in two formats: (i) mono-culture and (ii) co-culture:

#### i. Mono-culture experiments

We investigated the dose-dependent effects of RANKL (0–25 ng/mL) on osteoblastic activity in both HAECs and HASMCs over 72 hr, a treatment period and dose range previously employed by Davenport *et al*. [12,28] and shown to yield robust responses. In parallel experiments designed to examine the protective effects of TRAIL towards RANKL-induced signalling, cells were grown to confluency in standard 6-well culture dishes and treated for 72 hr with RANKL (5 or 25 ng/mL), in the absence and presence of 5 ng/mL TRAIL. Following all treatments, conditioned media (released protein) and cells (mRNA, cell protein) were routinely harvested for analysis. OPG and BMP-2 levels were monitored by ELISA, whilst ALP enzyme activity was monitored by QuantiChrom^™^ ALP Assay Kit (BioAssay Systems). mRNA expression levels for a range of genes linked to pro-calcific signalling (OPG, ALP, BMP-2, Runx2, and Sox9) were also assessed by qPCR. HAECs were exposed to TNF-α (100 ng/mL) as a positive control for BMP-2 production, whilst HASMCs were exposed to 10 mmol/L β-glycerophosphate as a positive control for induction of osteoblastic activity. All samples were stored at -80°C and assayed within three months.

#### ii. Co-culture experiments

The effect of HAEC paracrine signalling on HASMC osteoblastic activity was next investigated using a non-contact transwell co-culture model. Subluminal compartment; HASMCs were seeded at a density of 1.5x10^5^ cells per well into standard 6-well culture dishes and grown to confluency. Luminal compartment; HAECs were seeded into permeable (0.4 μm pore) transwell culture inserts (Merck Millipore, MA, USA) at a density of 2x10^5^ HAECs per insert and grown to confluency. Transwell inserts were then positioned into HASMC plate wells to establish co-culture conditions. A 50:50 mixture of HAEC:HASMC growth media was employed throughout the subsequent co-culture treatment period as previously described [12]. At the commencement of co-culture, HAECs were treated for 72 hr with RANKL (5 or 25 ng/mL), TRAIL (5 ng/mL), or both (5 or 25 ng/mL RANKL + 5 ng/mL TRAIL). In our view, this paradigm best represents the *in vivo* situation, whereby endothelial cells would be continuously exposed to these ligands, whilst engaging in ongoing paracrine signalling with the underlying medial smooth muscle cells. Post-treatment, HASMC-conditioned subluminal media and HASMCs (mRNA, cell protein) were harvested for analysis of ALP activity, OPG/BMP-2 levels and gene expression.

### Quantitative real-time PCR (qPCR)

Extraction of total RNA and preparation of cDNA was achieved using the TRIzol^™^ RNA extraction protocol (ThermoFisher Scientific) and the Applied Biosystems^™^ high-capacity cDNA reverse transcription kit (Thermo Fisher Scientific), respectively. Prior to cDNA preparation, all RNA samples were routinely pre-treated with DNase1 (Sigma-Aldrich). Amplification of target cDNA sequences using gene-specific primers was achieved using the LightCycler^®^96 real-time PCR system (Roche Diagnostics, West Sussex, UK). PCR reaction mixtures (10 μL) were as follows: 5 μL of FastStart Universal SYBR Green/Rox Mastermix (Roche Diagnostics), 1.5 μL of RNase-free water, 2.5 μL of cDNA, 0.5 μL each of 10 μmol/L forward and reverse primers. PCR reaction conditions were as follows: denaturation at 95°C for 10 min followed by 45 cycles of: (i) denaturation at 95°C for 10 sec, (ii) annealing at 59°C for 10 sec, and (iii) elongation at 72°C for 10 sec. Each cDNA sample was assayed in triplicate and results analysed by the comparative C_T_ method. GADPH was routinely used for normalization purposes. All primer pairs were designed for optimal efficiency according to MIQE guidelines and were pre-screened for correct product size (1% agarose gel electrophoresis). Primer pairs also underwent melt-curve analysis for confirmation of PCR product purity and detection of primer-dimers. GAPDH (238 bp): Forward 5’-gagtcaacggatttggtcgt-3’; Reverse 5’-ttgattttggagggatctcg-3’; ALP (293 bp): Forward 5’-gcctggctacaaggtggtg-3’; Reverse 5’-ggccagagcgagcagc-3’; Runx2 (315 bp): Forward 5’-ggtaccagatgggactgtgg-3’; Reverse 5’-gaggcggtcagagaacaaac-3’; Sox9 (85 bp): Forward 5’-agcgaacgcacatcaagac-3’; Reverse 5’-ctgtaggcgatctgttgggg-3’; OPG (241 bp): Forward 5’-ggcaacacagctcacaagaa-3’; Reverse 5’-ctgggtttgcatgcctttat-3’; BMP-2 (199 bp): Forward 5’-caagccaaacacaaacagcg-3’; Reverse 5’-ccaacgtctgaacaatggca-3’.

### Enzyme-linked immunosorbent assay (ELISA)

OPG and BMP-2 DuoSet^®^ ELISA Kits (R&D Systems) were employed as per manufacturer instructions (with minor volume modifications) to accurately measure absolute levels of OPG and BMP-2 in HAEC/HASMC lysates and conditioned media. Briefly, F96 Maxisorp™ Nunc-Immuno™ 96-well plates (Bio-Sciences Ltd., Dun Laoghaire, IRL) were coated with 50 μL/well of the provided capture antibody and incubated overnight at room temperature. The plate was then blocked by adding 150 μL of Reagent Diluent (1% high grade BSA) to each well and incubated for 1 hr at room temperature. HAEC/HASMC total protein lysates were routinely pre-diluted (depending on total protein levels) in Reagent Diluent for analysis of OPG and BMP-2 levels. HAEC-conditioned media samples remained undiluted, whilst HASMC-conditioned media samples were diluted for OPG analyses only. Dilution, when applied to lysate and media samples, was determined to work optimally within the 1:20 to 1:50 range. This ensured that microplate readings remained within the measurable linear range for ELISA analysis (46.9–3000 pg/mL for BMP-2; 62.5–2000 pg/mL for OPG). All samples and standards were subsequently assayed in duplicate at 50 μL/well and incubated for 2 hr at room temperature. Following sample incubation, 50 μL of the provided detection antibody was added to each well and then incubated for a further 2 hr at room temperature. Post-incubation, 50 μL of streptavidin-HRP was dispensed to each well and incubated for 20 min at room temperature in the dark. 50 μL of substrate solution was then added to each well and incubated for a further 20 min at room temperature in the dark. Reactions were terminated with the addition of 25 μL of stop solution (0.16 M sulfuric acid) to each well and the plate subsequently read at 570 nm with wavelength correction at 450 nm to account for plate optical imperfections. For normalization purposes, OPG and BMP-2 levels in protein lysates were routinely presented as pg/mg of total protein, whilst conditioned media levels were presented as pg/10^5^ cells.

### Alkaline phosphatase activity assay

Previous researchers have successfully employed the Quantichrom^™^ Kit (BioAssay Systems) to monitor ALP activity, a critical marker of osteoblastic activation [29]. For this paper, ALP activity was measured in vascular cell conditioned media and total protein lysate samples according to the manufacturer’s protocol. This colorimetric kinetic assay is based on the principle that ALP, if present in the sample, will hydrolyse p-Nitrophenyl phosphate (pNPP) into p-nitrophenol and phosphate, forming a yellow product with a maximum absorbance at 405 nm. For analyses, 50 μL of media or 5 μL of lysate (~5–10 μg protein) was added to each well. The working solution, consisting of assay buffer pH 10.5, 0.2 mol/L magnesium acetate and 1 mol/L pNPP, was then added to the sample to a total volume of 200 μL/well and subjected to a 4 min incubation at 37°C. The plate was read at both 0 and 4 min, with distilled H_2_O (negative control) and tartrazine (yellow liquid with a fixed absorbance) loaded for run calibration. All samples were assayed in duplicate. The subsequent colour change was measured via microplate reader at 405 nm and the absorbance at 4 min compared with baseline (0 min). ALP activity (IU/l) was then determined from the absorbance difference via formula provided by the manufacturer. Results are presented as fold change in ALP activity compared to control (0 ng/mL) to eliminate microplate reader variations.

### Statistical analysis

Results are expressed as mean±standard error of the mean (SEM). Experimental points were typically performed in triplicate with a minimum of three independent experiments (n = 3). Statistical comparisons between control and experimental groups was by ANOVA in conjunction with a Dunnett’s *post-hoc* test for multiple comparisons. A value of *P≤0.05 versus control was considered significant. A Student’s *t*-test was also routinely employed for pairwise comparisons (^δ^*P*≤0.05).

## Results

The current paper presents a detailed analyte profile of how RANKL elicits osteoblastic activation within vascular cells, adding greater depth to an earlier paper on this subject by our group [12]. Moreover, within this context, a central objective of the current paper was to address the specific hypothesis that TRAIL can elicit anti-calcific effects through blockade of RANKL action. A panel of analytes intrinsic to osteoblastic signalling was assayed and an overview of findings (many of which are novel) is highlighted in Table 1.

**Table 1:** Effect of RANKL±TRAIL (72 hr) upon key osteoblastic targets within HAEC and HASMC mono-cultures, as well as HAEC:HASMC co-cultures.

*Target*	*Sample Type*	HAEC			HASMC			HAEC:HASMC		
		R	T	R + T	R	T	R + T	R	T	R + T
**BMP-2**	*mRNA*	n.c.	n.c.	n.c.	n.c.	↓	n.c.	n.c.	↓	n.c.
	*Cellular Protein*	↑	n.c.	↑	↑	n.c.	n.c.	n.c.	n.c.	n.c.
	*Released Protein*	↑	n.c.	n.c.	n.c.	n.c.	n.c.	↑	n.c.	n.c.
**OPG**	*mRNA*	-	-	-	↓	n.c.	↓	↓	n.c.	n.c.
	*Cellular Protein*	n.c.	↑	n.c.	↑	↑	↑	↑	n.c.	n.c.
	*Released Protein*	n.c.	↑	n.c.	↓	n.c.	↓	↓	n.c.	↓
**ALP**	*mRNA*	n.c.	n.c.	-	n.c.	n.c.	n.c.	↑	↓	n.c.
	*Activity*	↑	↓	↓	↑	n.c.	↑	↑	n.c.	n.c.
**Runx2**	*mRNA*	n.c.	n.c.	-	↑	↓	n.c.	n.c.	n.c.	n.c.
**Sox9**	*mRNA*	-	-	-	n.c.	n.c.	n.c.	↑	n.c.	n.c.

Key: ALP, alkaline phosphatase; BMP-2, bone morphogenetic protein-2; OPG, osteoprotegerin; R, 25 ng/mL RANKL; Runx2, runt-related transcription factor 2; Sox9, sex-determining region-Y box 9; T, 5 ng/mL TRAIL; ↑, increase; ↓, decrease; n.c. no change;—not determined; Arrows reflect direction of change relative to untreated control.

It should be noted that receptors for RANKL (RANK) and TRAIL (DCR1, DCR2, DR4, and DR5) were all expressed at the mRNA level in both cell types (data not shown). Moreover, viability testing was incorporated into all studies with >90% viability routinely confirmed for the highest concentrations of RANKL and TRAIL tested (data not shown).

Given the high volume and diversity of data contained within Table 1, we have decided for reasons of narrative focus to selectively highlight results for just one characteristic target within HAEC (BMP-2) and HASMC (OPG) mono-culture studies. In this respect, HAEC-derived BMP-2 has previously been shown to cause osteoblastic activation of underlying HASMCs [12], whilst HASMCs are a major producer of anti-calcific OPG within the vessel wall. For our co-culture studies however, we present a broader analysis to highlight the influence of RANKL and TRAIL on several key factors (OPG, ALP, Runx2, Sox9).

### Direct effects of RANKL±TRAIL on HAEC BMP-2 levels

**Fig 1:**
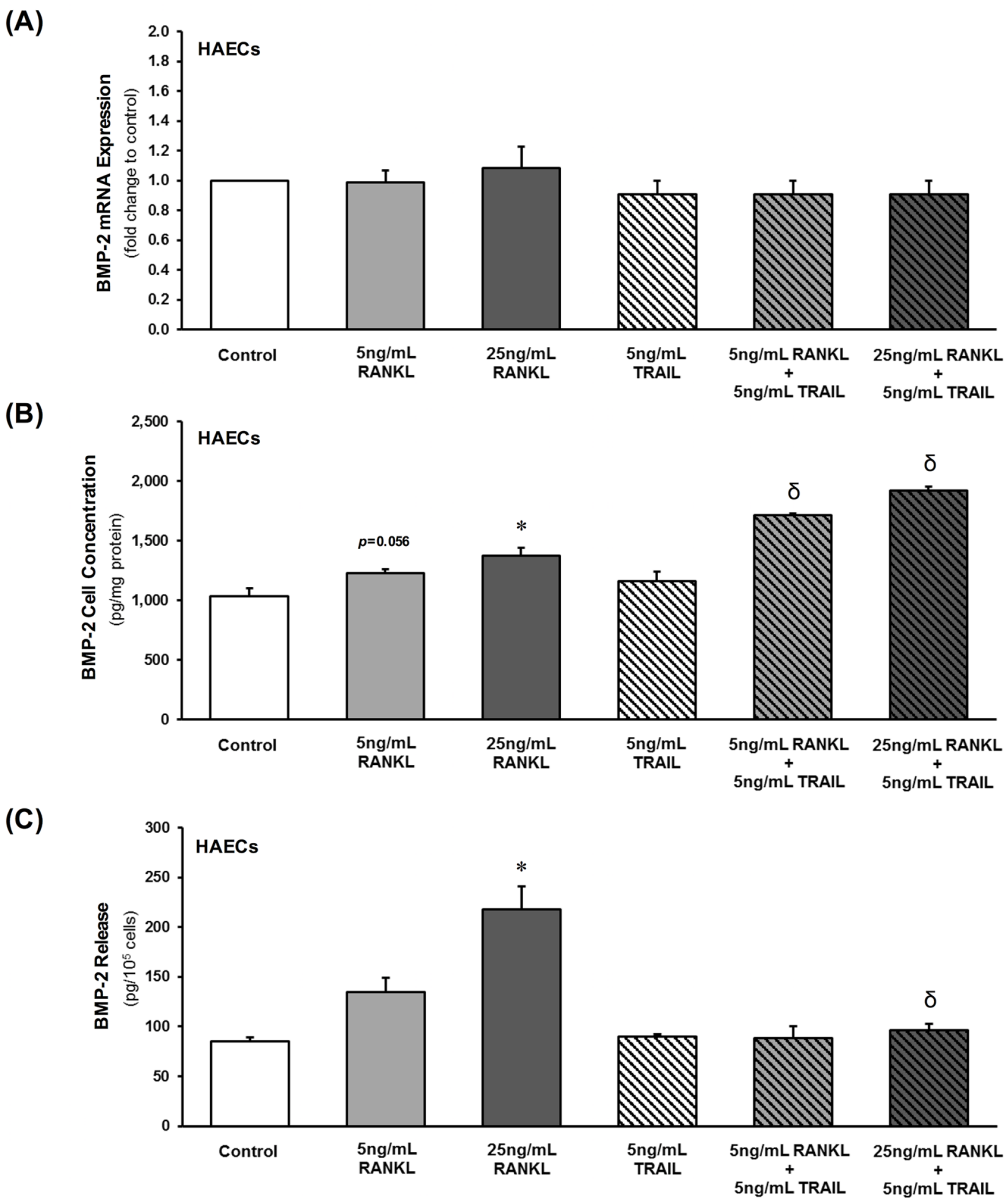
Direct effects of RANKL±TRAIL on BMP-2 levels in HAECs. HAECs were treated for 72 hr with RANKL (0–25 ng/mL) in the absence and presence of TRAIL (5 ng/mL) and then analyzed by qPCR for (**A**) BMP-2 mRNA. Cells and conditioned media were also harvested for ELISA analysis of (**B**) BMP-2 cellular protein and (**C**) released BMP-2, respectively. *P≤0.05 versus 0 ng/mL RANKL; ^δ^*P*≤0.05 versus corresponding 5 and 25 ng/mL RANKL treatments.

In initial studies, the effects on BMP-2 expression and release following HAEC exposure to RANKL (0–25 ng/mL, 72 hr) were assayed. RANKL had no effects on BMP-2 mRNA levels (Fig 1A) and only moderate effects on BMP-2 protein levels (Fig 1B). Most notably however, RANKL was observed to increase BMP-2 release from HAECs in a dose-dependent manner (Fig 1C). Furthermore, inclusion of TRAIL at 5 ng/mL was found to completely block the effects of RANKL on BMP-2 release from HAECs, whilst simultaneously increasing intracellular accumulation of BMP-2 (Fig 1B and 1C).

### Direct effects of RANKL±TRAIL on HASMC OPG levels

**Fig 2:**
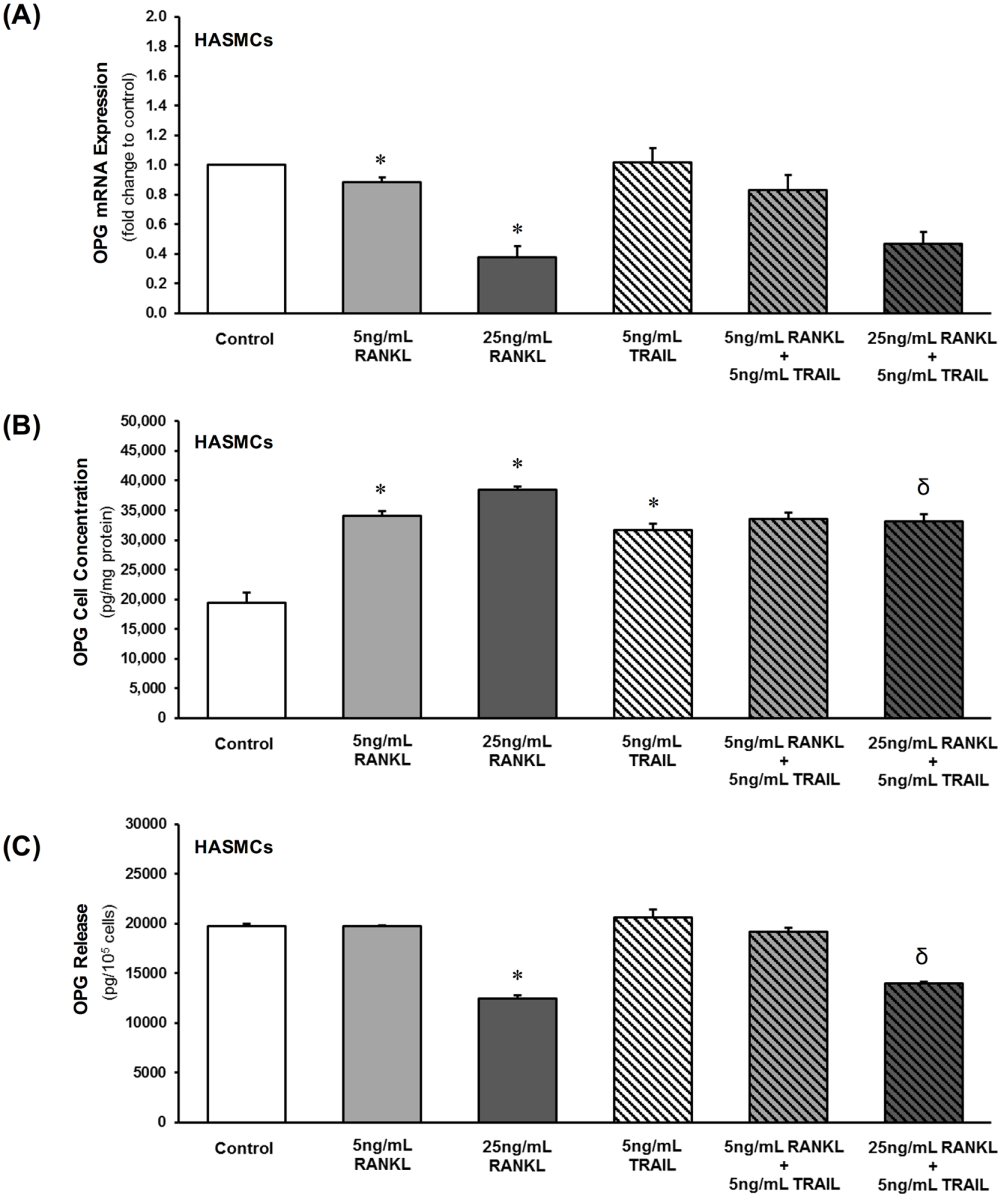
Direct effects of RANKL±TRAIL on OPG levels in HAECs. HAECs were treated for 72 hr with RANKL (0–25 ng/mL) in the absence and presence of TRAIL (5 ng/mL) and then investigated by qPCR for (**A**) OPG mRNA. Cells and conditioned media were also harvested for ELISA analysis of (**B**) OPG cellular protein and (**C**) released OPG, respectively. *P≤0.05 versus 0 ng/mL RANKL (or control); ^δ^*P*≤0.05 versus 25 ng/mL RANKL.

The effects on OPG expression and release following HASMC exposure to RANKL (0–25 ng/mL, 72 hr) were investigated. RANKL decreased OPG mRNA levels (Fig 2A). It also increased cellular OPG protein levels in HASMCs in a dose-dependent manner, whilst decreasing OPG release (Fig 2B and 2C). Inclusion of 5 ng/mL TRAIL had no effect on RANKL-induced changes in either OPG mRNA or release. However, it appeared to prevent RANKL-induced increases in cellular OPG protein (when baseline effects of 5 ng/mL TRAIL are taken into account) (Fig 2B).

### Paracrine effects of RANKL±TRAIL on HASMC osteoblastic activation in a co-culture model

**Fig 3:**
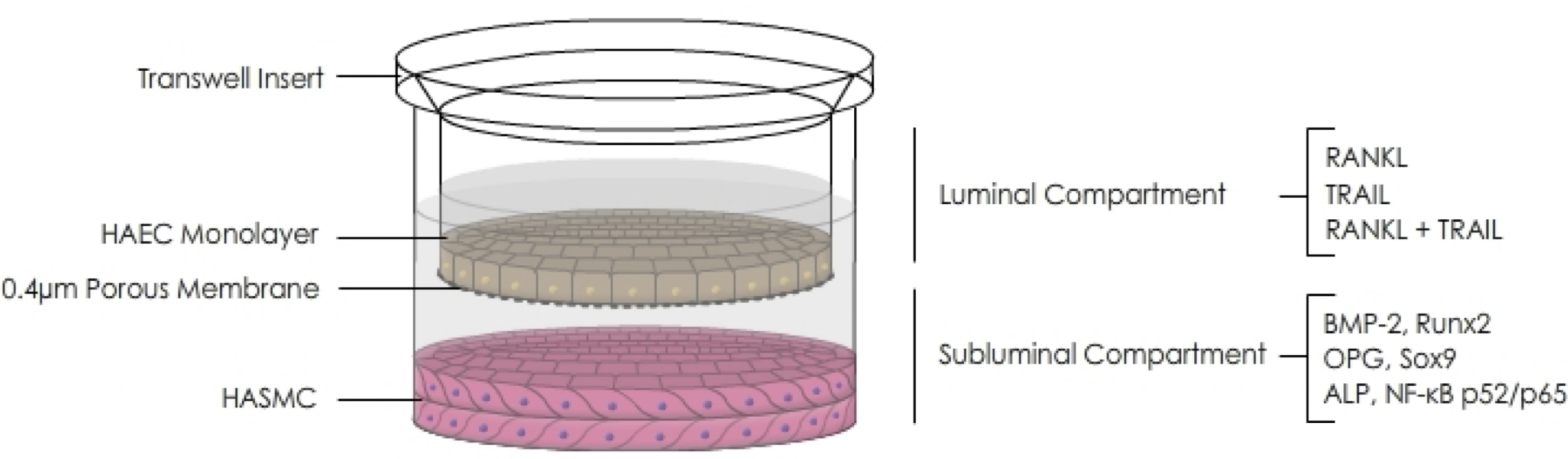
Schematic of the HAEC:HASMC transwell co-culture model. HAECs within the luminal compartment were treated for 72 hr with RANKL (0–25 ng/mL) in the absence and presence of TRAIL (5 ng/mL). Within the subluminal compartment, HASMCs were then analyzed for key targets (OPG, ALP, Runx2, Sox9, BMP-2.

**Fig 4:**
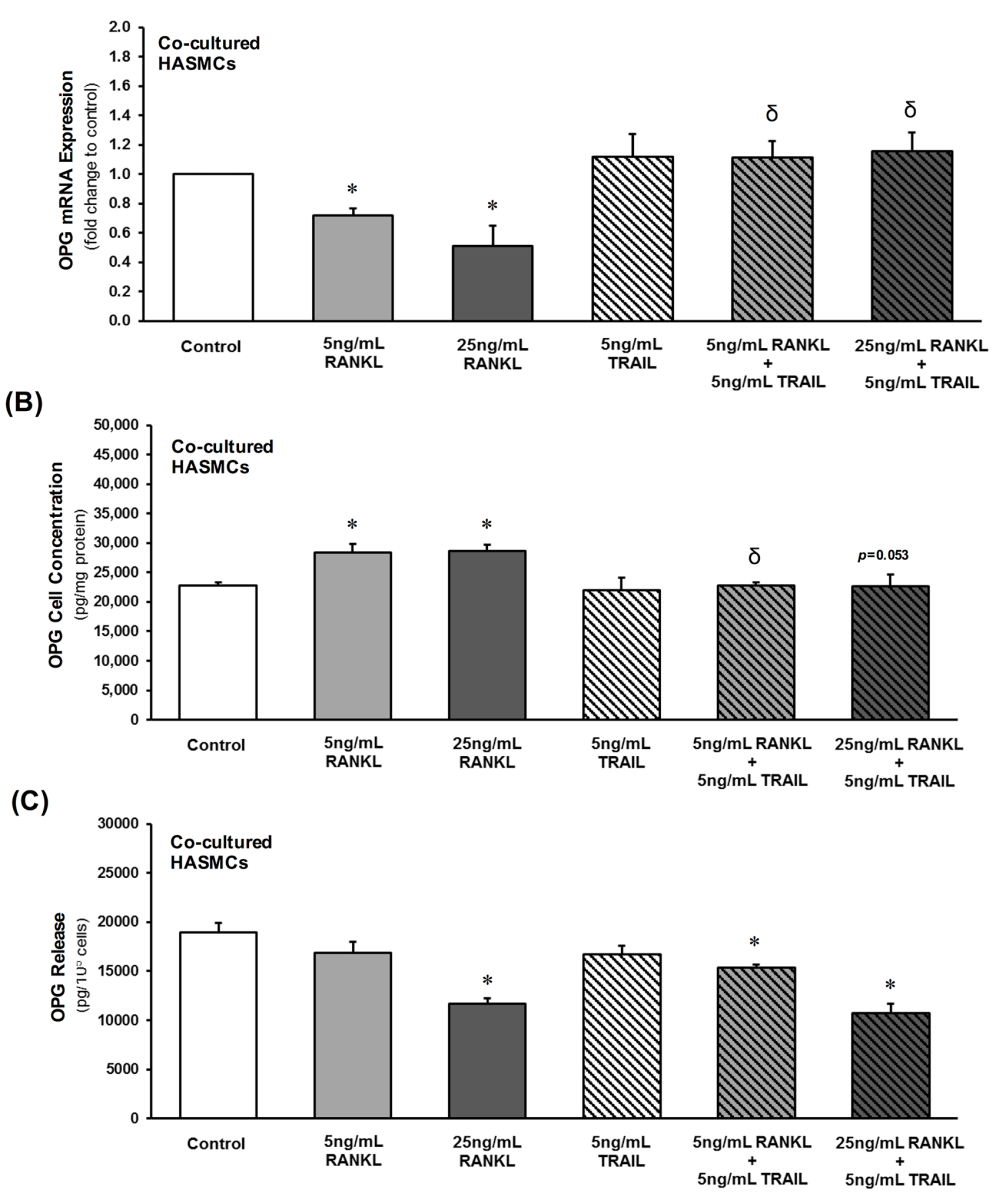
Effects of RANKL±TRAIL on HASMC OPG levels within a HAEC:HASMC co-culture model. HAECs within the luminal compartment were treated for 72 hr with RANKL (0–25 ng/mL) in the absence and presence of TRAIL (5 ng/mL). Subluminal HASMCs were then analyzed by qPCR for (**A**) OPG mRNA. Subluminal HASMCs and conditioned media were also harvested for ELISA analysis of (**B**) OPG cellular protein and (**C**) released OPG, respectively. *P≤0.05 versus 0 ng/mL RANKL (or control); ^δ^*P*≤0.05 versus corresponding 5 and 25 ng/mL RANKL treatments.

A transwell co-culture model comprising HAECs in the luminal compartment and HASMCs in the subluminal compartment was set up (Fig 3). HAECs were then treated with RANKL (0–25 ng/mL, 72 hr) and their subsequent paracrine effect upon osteoblastic signalling within the underlying HASMCs was investigated. RANKL treatment of HAECs decreased HASMC OPG mRNA levels and release in a dose-dependent manner, whilst increasing intracellular HASMC OPG protein levels (Fig 4A–4C). Importantly, inclusion of 5 ng/mL TRAIL with RANKL in the HAEC compartment completely reversed the RANKL-induced changes to both OPG mRNA and intracellular protein levels within the underlying HASMC layer (Fig 4A and 4B).

**Fig 5:**
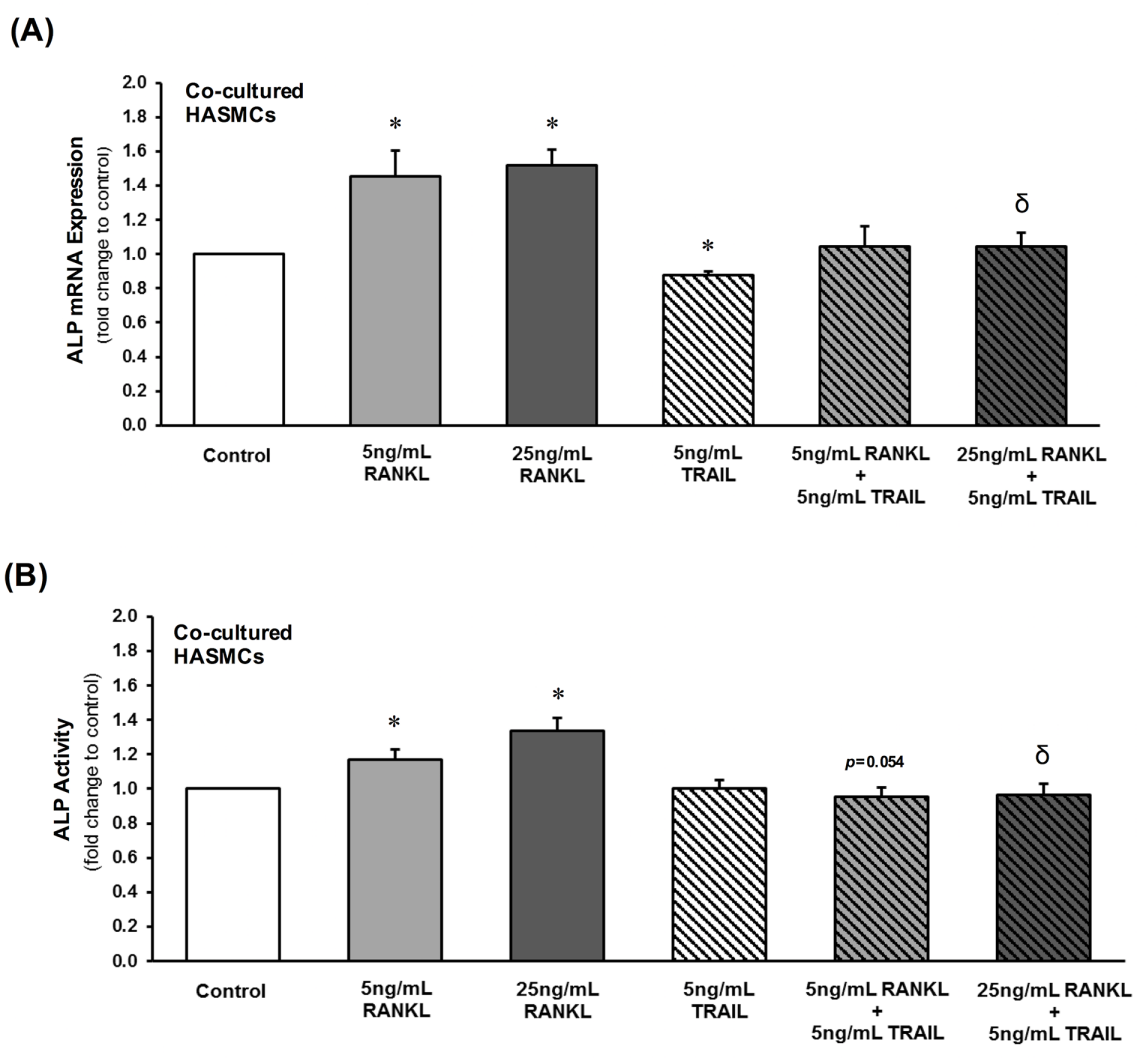
Paracrine effects of RANKL±TRAIL on HASMC ALP levels within a HAEC:HASMC co-culture model. HAECs within the luminal compartment were treated for 72 hr with RANKL (0–25 ng/mL) in the absence and presence of TRAIL (5 ng/mL). Within the subluminal compartment, HASMCs were then harvested and analyzed for (**A**) ALP mRNA and (**B**) ALP enzymatic activity by qPCR and ELISA, respectively. *P≤0.05 versus 0 ng/mL RANKL (or control); ^δ^*P*≤0.05 versus 25 ng/mL RANKL.

**Fig 6:**
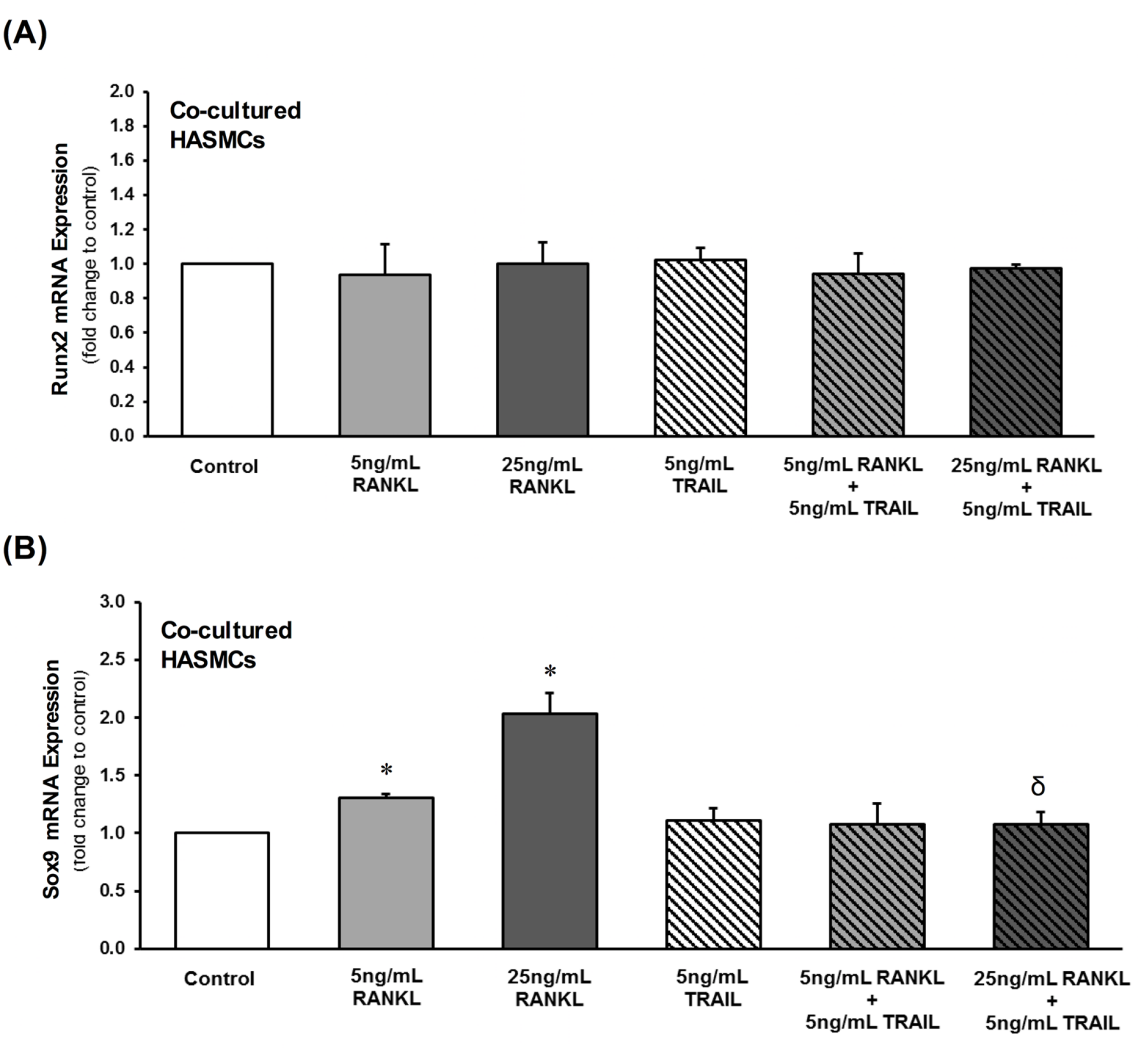
Paracrine effects of RANKL±TRAIL on HASMC osteoblastic transcription factors within a HAEC:HASMC co-culture model. HAECs within the subluminal compartment were treated for 72 hr with RANKL (0–25 ng/mL) in the absence and presence of TRAIL (5 ng/mL). Within the subluminal compartment, HASMCs were then analyzed by qPCR for (**A**) Runx2 and (**B**) Sox9 mRNA. *P≤0.05 versus 0 ng/mL RANKL (or control); ^δ^*P*≤0.05 versus 25 ng/mL RANKL.

In addition to OPG, other osteoblastic events in HASMCs were examined. RANKL treatment of HAECs dose-dependently increased HASMC ALP mRNA and enzymatic activity levels (Fig 5A and 5B), as well as Sox9 (but not Runx2) mRNA levels (Fig 6A and 6B). Importantly, inclusion of 5 ng/mL TRAIL with RANKL in the HAEC compartment completely reversed the RANKL-induced changes to both ALP (Fig 5A and 5B) and Sox9 (Fig 6B) within the underlying subluminal HASMC layer.

## Discussion

Vascular calcification (VC) is a major risk factor for elevated CV morbidity and mortality associated with aging and systemic diseases (e.g. diabetes mellitus, chronic kidney disease). Understandably, a deeper knowledge of the mechanisms underpinning VC development is essential if therapeutic interventions are to be devised. The pivotal involvement of RANKL, OPG and TRAIL within osteoblastic events leading to VC, regulatory proteins known to be active within the vasculature, has been highlighted within the scientific literature [for review, see 8], although much still remains to be discovered about their integrative roles. In the present study, we set out to broaden our fundamental knowledge of how RANKL influences osteoblastic signalling at the immediate endothelial and smooth muscle cell levels, as well as across a more physiologically relevant paracrine co-culture model of these two cell types. Moreover, we test the hypothesis that key osteoblastic signalling events promoted in vascular cells by RANKL can be attenuated by TRAIL. In support of this hypothesis, a growing body of evidence now points to the vasoprotective effects and therapeutic benefits of TRAIL. Moreover, recent *in vivo* studies have demonstrated how TRAIL deficiency can lead to accelerated calcification in ApoE^-/-^ mice [25], whilst earlier *in vitro* studies in human and murine pre-osteoclast cells have reported on the ability of TRAIL to counteract RANKL-mediated signalling [26,27].

In initial experiments, we investigated the direct effects of RANKL on HAECs in mono-culture with particular focus on BMP-2, a pro-osteogenic protein known to elicit osteoblastic activation in underlying HASMCs [1,12]. RANKL treatment of HAECs elevated BMP-2 cellular levels (slightly) and BMP-2 release (substantially). By contrast, there was no significant effect of RANKL on BMP-2 mRNA expression. In support of our hypothesis, co-incubation of HAECs with TRAIL completely blocked the RANKL-induced release of BMP-2, but not the RANKL-induced elevation in cellular concentrations of BMP-2 (in fact, cellular concentrations of BMP-2 actually rose slightly). Thus, we suspect that the blockade of RANKL-induced BMP-2 secretion by TRAIL may be causing an accumulation of BMP-2 within the cell.

Interestingly, separate control HAEC experiments demonstrated how TRAIL could dose-dependently increase the expression and release of OPG (S1 Fig), a decoy receptor for both RANKL and TRAIL. Given the statistically negligible levels of OPG release induced by 5 ng/mL TRAIL (the concentration routinely used in our co-incubation studies with RANKL), it is unlikely that any attenuation of RANKL action by TRAIL observed in these studies can be attributed to elevated RANKL inactivation arising from induced release of OPG decoy receptor. It is also worth noting that, to our knowledge, there are no existing papers proposing a mechanism for TRAIL-dependent blockade of RANKL action in endothelial cells. One earlier study by Osako and co-workers has demonstrated that estrogen, acting through estrogen receptor alpha, can block RANKL-mediated production of BMP-2 in HAECs via a mechanism involving dephosphorylation of SMAD-1/5/8 and increased expression of the calcification inhibitor, matrix Gla protein (MGP) [13]. There is however no current evidence within the literature linking TRAIL action to these signalling pathways within the vasculature.

We next investigated the direct effects of RANKL on HASMCs with particular focus on OPG, an anti-calcific ligand produced and released in high quantities by HASMCs. We noted that RANKL treatment of HASMCs decreased OPG mRNA expression and release. These novel findings indicate how the osteoblastic effects of RANKL in HASMCs may possibly be achieved in-part through suppression of the release of its endogenous decoy receptor (and most likely also explain the parallel OPG intracellular accumulation). This conclusion is consistent with the recent work of Callegari *et al*., who show that RANKL-induced calcification is enhanced in murine VSMCs isolated from OPG^-/-^ ApoE^-/-^ mice relative to WT mice [30], and also of Panizo *et al*., who demonstrate how the osteoblastic effects of RANKL in VSMCs can be abolished by co-incubation with exogenous OPG [10]. Importantly, co-incubation of HASMCs with TRAIL had no effect on RANKL-induced suppression of OPG, nor indeed did TRAIL have any effect on RANKL-induced increases in activation of ALP (Table 1) or NF-κB (data not shown) in HASMCs. Also noteworthy from this series of experiments, RANKL treatment of HASMCs was seen to increase OPG cellular concentrations, a phenomenon that could be blocked by co-incubation of cells with TRAIL. In our view, it is unlikely that this effect of TRAIL is attributable to either increased OPG release (Fig 2C) or decreased OPG mRNA translation (Fig 2B). It may be possible however that the specific combination of RANKL with TRAIL may be increasing ubiquitination and proteasomal turnover of OPG protein within the cell, thereby decreasing RANKL-induced cellular OPG levels.

In order to more accurately recapitulate *in vitro* the paracrine signalling axis that exists within the vessel wall between endothelial and smooth muscle cells, we next investigated how RANKL treatment of HAECs could influence osteoblastic events within underlying HASMCs using a non-contact transwell co-culture model. In this respect, RANKL treatment of HAECs was shown to elicit a range of osteoblastic responses in underlying HASMCs, including reduction in OPG levels coupled with elevations in ALP and Sox9 (a chondrocytic differentiation factor that has been shown to occur in VSMC calcification, and that is associated with many of the same gene/protein alterations associated with osteogenic differentiation [31–33]). Most importantly for our hypothesis, these HASMC responses could all be completely blocked by co-incubation of RANKL with TRAIL in the HAEC compartment. Moreover, there was no evidence of significant RANKL/TRAIL diffusion from the luminal to subluminal compartments across the endothelial monolayer. Our findings with co-culture therefore clearly support our hypothesis and highlight for the first time the ability of TRAIL to directly counteract specific signalling actions of RANKL particularly at the endothelial cell level, with putative consequences for osteoblastic activation in the underlying smooth muscle cells. It can be noted that the application of co-culture models to successfully demonstrate the paracrine contribution of various cell types to VSMC calcification has been highlighted previously [34,35]. In particular, the importance of paracrine signalling is also highlighted in a recent paper by Davenport *et al*. [12], who showed that conditioned media harvested from the subluminal aspect of RANKL-treated HAECs, when incubated with independent HASMC reporter cultures, led to an increase in ALP levels in HASMCs. These authors also demonstrated that this effect could be blocked by noggin, a specific neutralizing ligand for the released BMP-2 present within the HAEC conditioned media. In this respect, our current paper further reinforces the physiological significance of this BMP-2 observation made by Davenport *et al*. by demonstrating that the RANKL-induced release of BMP-2 from HAECs could be completely blocked by TRAIL. Moreover, in separate investigations we have shown how treatment of HASMCs with recombinant BMP-2 can in-turn induce expression of a range of osteogenic markers such as ALP, Sox9, and Runx2 (S2 Fig). Furthermore, we have demonstrated using our HAEC:HASMC co-culture model that when HAECs are treated with RANKL, the expected induction of underlying HASMC ALP levels can be almost completely blocked by inclusion of BMP-2-neutralizing noggin within the subluminal compartment (S3 Fig). These data have led us to propose that TRAIL can block key signalling actions of RANKL on endothelial cells, including BMP-2 release, resulting in attenuation of its associated osteogenic signalling actions upon underlying smooth muscle cells. Whilst beyond the scope of the current paper, a deeper clarification of this paracrine signalling relationship constitutes an ongoing goal of this research, with our preliminary studies clearly pointing to the ability of TRAIL to block RANKL-dependent activation of the non-canonical NF-κB/p52 pathway at the endothelial cell level (data not shown).

Whilst our general focus in this paper has been the osteoblastic signalling that ultimately leads to calcification, considerable efforts were also made to investigate the impact of RANKL (±TRAIL) on actual ‘end-point’ calcification levels in our HASMCs. In initial experiments, β-glycerophosphate treatment was found to induce HASMC osteoblastic signalling (S4 Fig), confirming the responsiveness of our cells to an established mineralizing stimulus [36]. Moreover, when HASMCs were incubated in osteoblastic differentiation medium for 21 days (S5 Fig), we noted the characteristic activation of early calcific markers such as ALP and bone sialoprotein (BSP), in conjunction with the suppression of smooth muscle alpha 2 actin (ACTA2) and transgelin (TAGLN) [37,38] (S1 Supplementary Methods). Following 21 days of osteoblastic differentiation however, we did not see activation of osteocalcin (OCN), a late-stage calcification marker in VSMCs and osteoblasts that is closely linked to the mineralisation process [39–41] and unsurprisingly therefore, did not see positive alizarin red staining for calcium in our differentiated HASMCs (S7A Fig), even with RANKL treatment. By way of a positive methodological control, parallel incubation of mouse MC3T3-E1 pre-osteoblasts in osteoblastic differentiation medium for 21 days induced significant elevation in levels of all signalling markers (S6 Fig) and yielded positive alizarin red staining for calcium deposition (S7 Fig) (S1 Supplementary Methods). We note previous studies that have achieved end-point calcification and positive alizarin red staining in VSMCs have specifically employed ‘calcifying’ VSMCs that have either been clonally selected for the mineralizing phenotype [39] or were isolated directly from calcified vessels [40] with subsequent multi-passage culturing in high calcium medium. In this respect, the apparent lack of end-point calcification in our HASMC cultures after 21 days’ of osteoblastic differentiation may be attributable to a combination of culture heterogeneity and our deliberate exclusion of calcium from the standard HASMC culture medium. To expand further upon this conclusion, it is noteworthy that the issue of culture heterogeneity has previously been raised by Olesen *et al*. in their recent thorough exploration of VC in VSMCs, wherein they propose that calcium deposition is an “unreliable endpoint” with a high degree of variability between vascular cell donors [42]. Moreover, the use of calcium- and phosphate-supplemented osteogenic media to help differentiate VSMCs towards end-point calcium deposition can be regarded as a potential confounding factor within experiments as these elements can directly induce mineralization, thereby potentially obscuring the pro-calcific effects of other agents under investigation (e.g. RANKL).

In summary, the present study has comprehensively profiled a broad range of osteoblastic signalling effects of RANKL in HAECs and HASMCs using both mono- and co-culture models, adding fresh detail to an earlier study on this subject by our group [12] and demonstrating that RANKL can act on both cell types either directly or via paracrine signalling axes. Within this context, we also present clear evidence that TRAIL can block key RANKL signalling effects, particularly at the endothelial cell level, with consequences for underlying smooth muscle cells. To our knowledge, this is the first time this specific inhibitory relationship between RANKL and TRAIL has been investigated and confirmed within vascular cell models, providing fresh evidence of TRAIL’s vasoprotective potential.

## Supporting information

S1 Fig Effects of TRAIL on calcification signals in HAECs. HAECs were treated for 72 hr with TRAIL (0–50 ng/mL) and analyzed by ELISA for OPG levels in conditioned media (**A**) and in cell lysates (**B**). *P≤0.05 versus 0 ng/mL TRAIL.
(TIF)

S2 Fig Effects of BMP-2±Noggin on osteoblastic markers in HASMCs. Cells were treated for 72 hr with BMP-2 (5 ng/mL) in the absence and presence of Noggin (100 ng/mL), and then analyzed by qPCR for (**A**) ALP, (**B**) Sox9, and (**C**) Runx2 mRNA. *P≤0.05 versus 0 ng/mL BMP-2 (or control). ^δ^*P*≤0.05 versus 5 ng/mL BMP-2. Note: Recombinant human BMP-2 (Catalog Number: PHC7145) was sourced from ThermoFisher Scientific (Waltham, MA, USA). Recombinant human Noggin (Catalog Number: 6057-NG) was sourced from R&D Systems (Minneapolis, MN, USA).
(TIF)

S3 Fig Effects of RANKL±Noggin on HASMC ALP levels within a HAEC:HASMC co-culture model. HAECs within the luminal compartment were treated for 72 hr with RANKL (0–25 ng/mL) in the absence and presence of Noggin (100 ng/mL). Within the subluminal compartment, HASMCs were then analyzed by qPCR for (**A**) ALP mRNA, whilst subluminal conditioned media was harvested and analyzed for (**B**) ALP enzymatic activity. *P≤0.05 versus 0 ng/mL RANKL (or control); ^δ^*P*≤0.05 versus corresponding 5 or 25 ng/mL RANKL.
(TIF)

S4 Fig Effects of β-glycerophosphate on osteoblastic marker levels in HASMCs. Cells were treated for 72 hr with β-glycerophosphate (10 mM) and then analyzed by qPCR for (**A**) ALP, (**B**) BMP-2, (**C**) Runx2 and (**F**) Sox9 mRNA. HASMCs were also harvested and analyzed for (**D**) ALP activity and (**E**) BMP-2 levels using enzyme assay and ELISA, respectively. *P≤0.05 versus 0 mM β-glycerophosphate.
(TIF)

S5 Fig Effects of osteoblastic differentiation on marker levels in HASMCs. Cells were treated for 21 days with either standard media or osteoblastic differentiation media^**γ**^ and then analyzed by qPCR for (**B**) BSP, (**C**) OCN, (**D**) ACTA2 and (**E**) TAGLN mRNA. HASMCs were also harvested and analyzed for (**A**) ALP enzymatic activity. *P≤0.05 versus Undifferentiated. Key: BSP, bone sialoprotein; OCN, osteocalcin; ACTA2, smooth muscle alpha 2 actin; TAGLN, transgelin. ^**γ**^Osteoblastic differentiation media details: Minimum essential medium eagle (Sigma-Aldrich, M8042) supplemented with 0.292 g/L L-glutamine (Sigma-Aldrich, G6392), 100 nM dexamethasone (Sigma-Aldrich, D4902), 50 μM ascorbic acid 2-phosphate (Sigma-Aldrich, 49752), 10 mM β-glycerophosphate (Sigma-Aldrich, G9422), 10% FBS (Sigma-Aldrich, F6178) and 1% Pen/Strep (Sigma-Aldrich, P4333).
(TIF)

S6 Fig Effects of osteoblastic differentiation on marker levels in mouse MC3T3-E1 pre-osteoblasts. Cells were treated for 21 days with either standard media or osteoblastic differentiation media and then analyzed by qPCR for (**A**) BSP, (**B**) ALP, (**C**) Sox9, (**D**) OCN and (**E**) Runx2 mRNA. Conditioned media was also harvested and analyzed for (**F**) ALP enzymatic activity. *P≤0.05 versus Undifferentiated. ^**γ**^Osteoblastic differentiation media details: As outlined in S5 Fig above.
(TIF)

S7 Fig Effects of osteoblastic differentiation on calcification levels in mouse MC3T3-E1 pre-osteoblasts. Cells were treated for 21 days with either standard media or osteoblastic differentiation media* and then analyzed by alizarin red staining for calcium deposition. Light microscopy showing alizarin red staining levels in MC3T3-E1 (but not in HASMCs) under 10X magnification (**A**) and for MC3T3-E1 cells in 6-well plates (**B**). Extraction of alizarin red dye from undifferentiated and differentiated MC3T3-E1 cells and quantitative spectroscopic analysis is also shown in the histogram (**B**, lower). *P≤0.05 versus Undifferentiated. ^**γ**^Osteoblastic differentiation media details: As outlined in S5 Fig above.
(TIF)

S1 Supplementary Methods This includes methods specifically associated with supporting figures (S5–S7 Figs) and includes culture of MC3T3-E1 pre-osteoblasts, alizarin red staining protocols, and qPCR primer sequences for human (BSP, OCN, ACTA2, TAGLN) and murine (BSP, OCN) calcification markers. (PDF)
