# Supplementary figures and images for: TRAIL attenuates RANKL-mediated osteoblastic signalling in vascular cell mono-culture and co-culture models
Source: PLoS One. 2017 Nov 16;12(11):e0188192. doi: 10.1371/journal.pone.0188192 (PMC5690591; doi:10.1371/journal.pone.0188192)

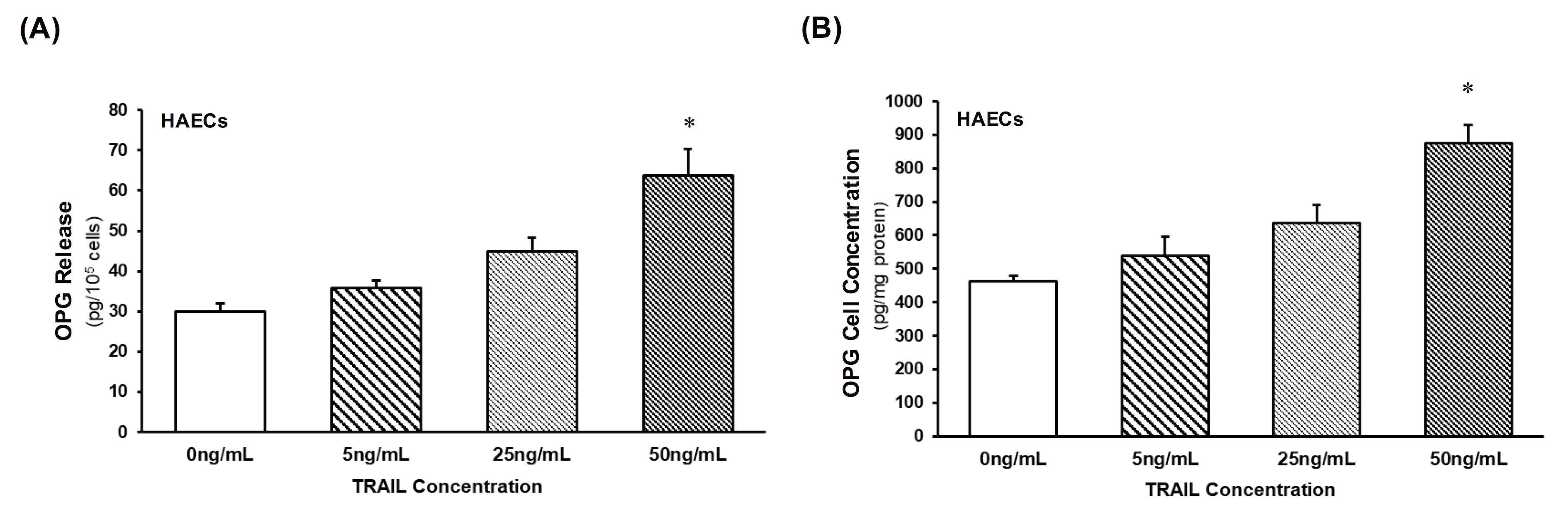

Supplement: S1 Fig — HAECs were treated for 72 hr with TRAIL (0–50 ng/mL) and analyzed by ELISA for OPG levels in conditioned media (A) and in cell lysates (B). *P≤0.05 versus 0 ng/mL TRAIL. (TIF) [file pone.0188192.s001.tif]

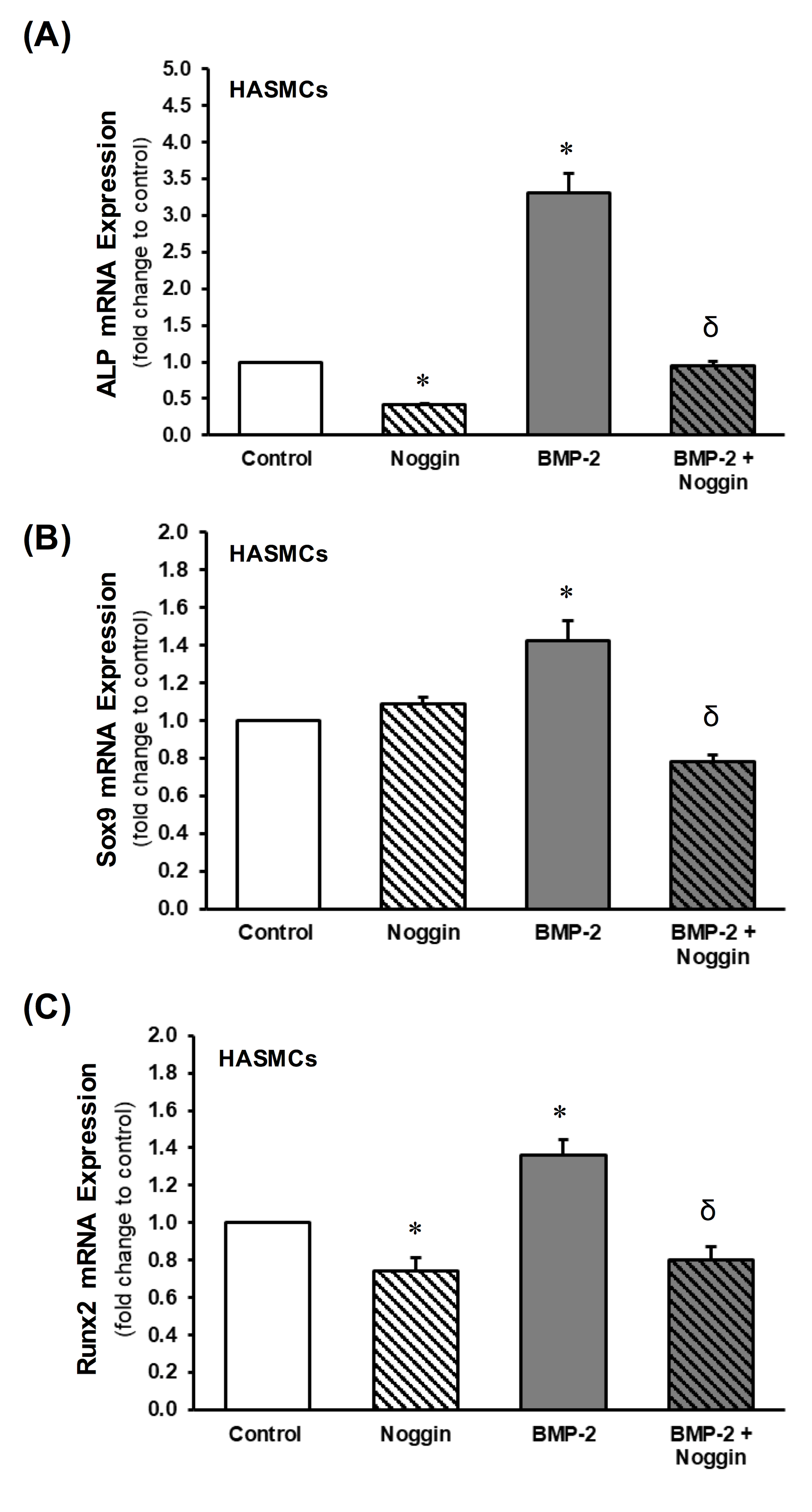

Supplement: S2 Fig — Cells were treated for 72 hr with BMP-2 (5 ng/mL) in the absence and presence of Noggin (100 ng/mL), and then analyzed by qPCR for (A) ALP, (B) Sox9, and (C) Runx2 mRNA. *P≤0.05 versus 0 ng/mL BMP-2 (or control). δP≤0.05 versus 5 ng/mL BMP-2. Note: Recombinant human BMP-2 (Catalog Number: PHC7145) was sourced from ThermoFisher Scientific (Waltham, MA, USA). Recombinant human Noggin (Catalog Number: 6057-NG) was sourced from R&D Systems (Minneapolis, MN, USA). (TIF) [file pone.0188192.s002.tif]

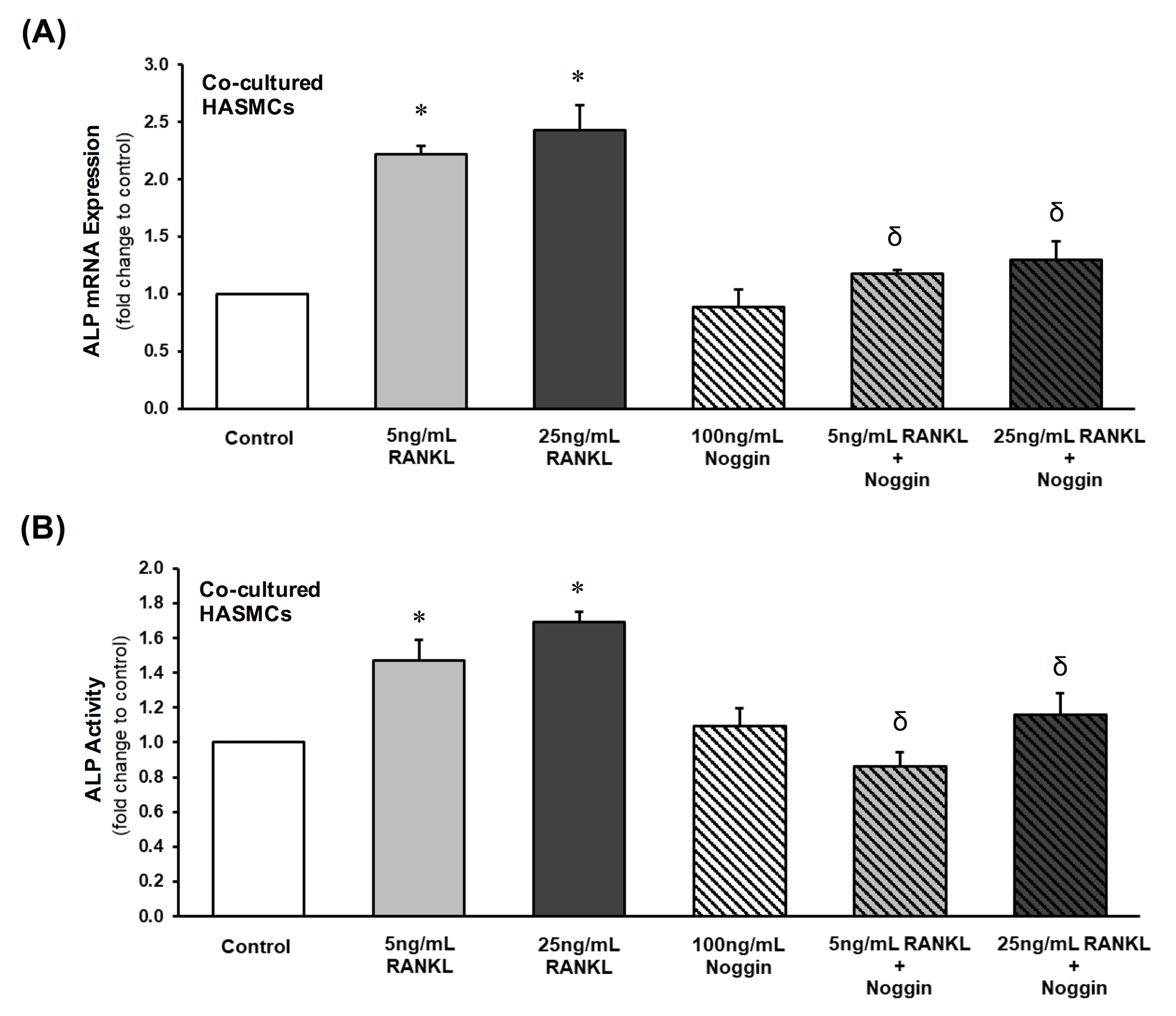

Supplement: S3 Fig — HAECs within the luminal compartment were treated for 72 hr with RANKL (0–25 ng/mL) in the absence and presence of Noggin (100 ng/mL). Within the subluminal compartment, HASMCs were then analyzed by qPCR for (A) ALP mRNA, whilst subluminal conditioned media was harvested and analyzed for (B) ALP enzymatic activity. *P≤0.05 versus 0 ng/mL RANKL (or control); δP≤0.05 versus corresponding 5 or 25 ng/mL RANKL. (TIF) [file pone.0188192.s003.tif]

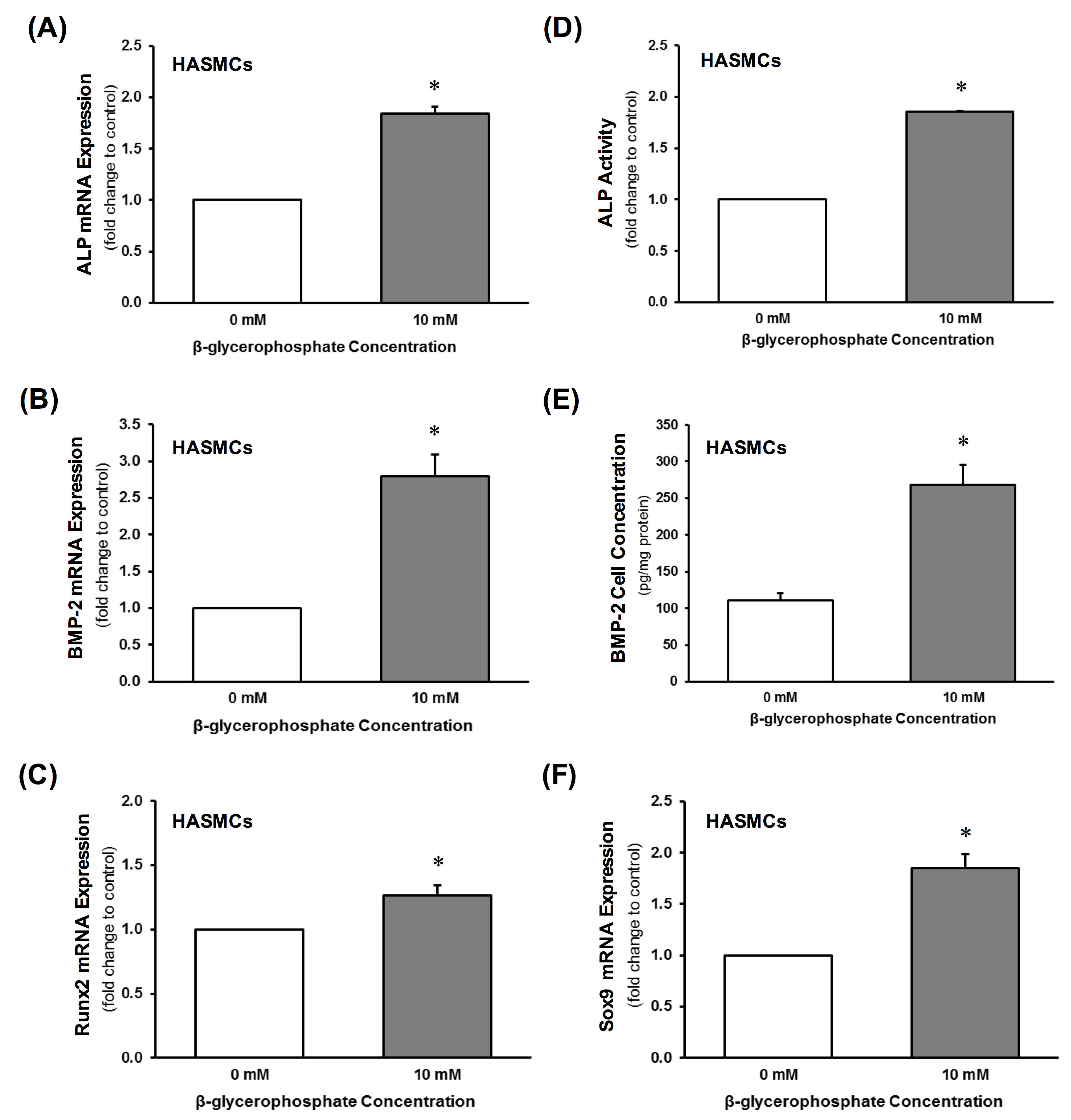

Supplement: S4 Fig — Cells were treated for 72 hr with β-glycerophosphate (10 mM) and then analyzed by qPCR for (A) ALP, (B) BMP-2, (C) Runx2 and (F) Sox9 mRNA. HASMCs were also harvested and analyzed for (D) ALP activity and (E) BMP-2 levels using enzyme assay and ELISA, respectively. *P≤0.05 versus 0 mM β-glycerophosphate. (TIF) [file pone.0188192.s004.tif]

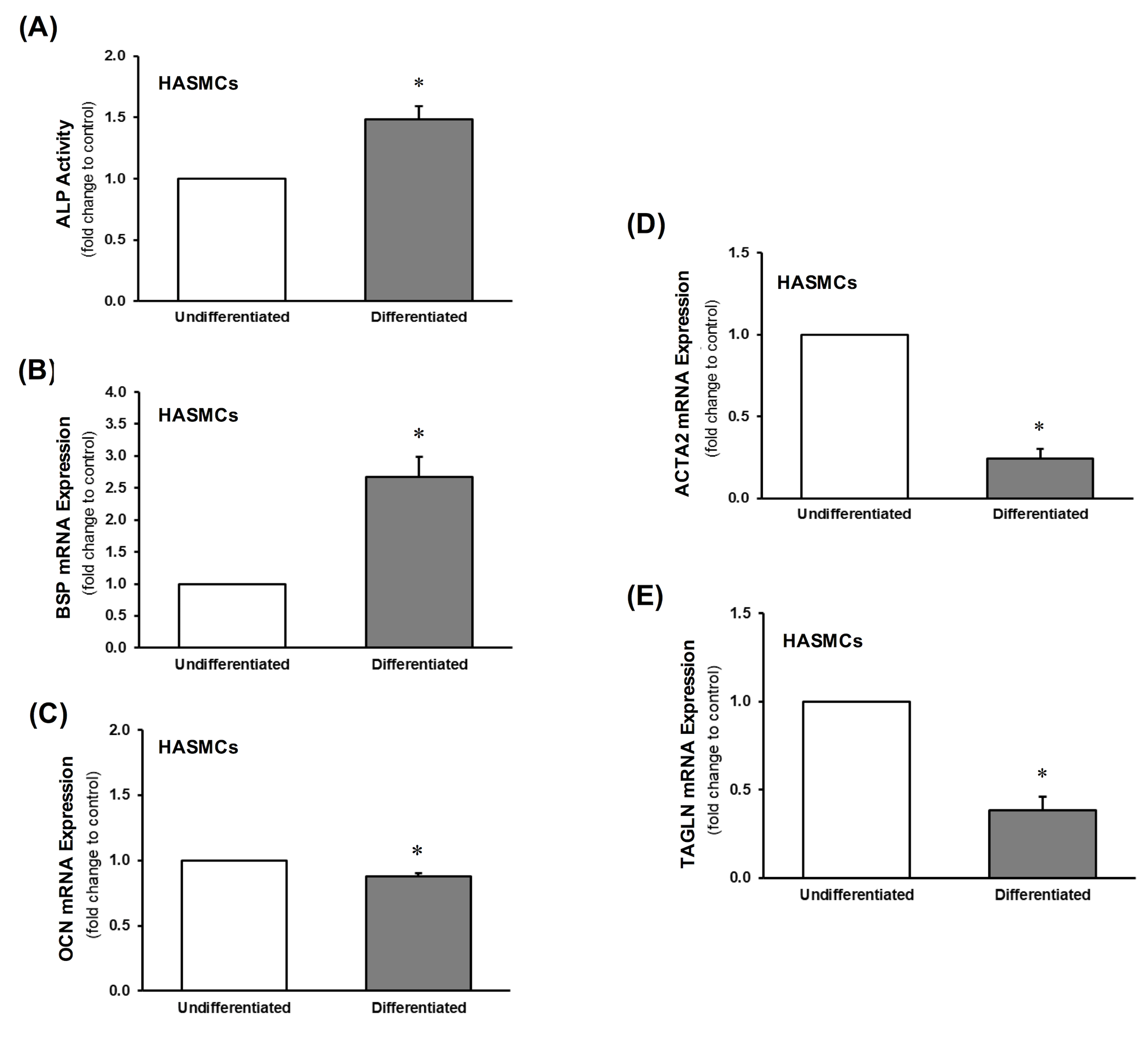

Supplement: S5 Fig — Cells were treated for 21 days with either standard media or osteoblastic differentiation mediaγ and then analyzed by qPCR for (B) BSP, (C) OCN, (D) ACTA2 and (E) TAGLN mRNA. HASMCs were also harvested and analyzed for (A) ALP enzymatic activity. *P≤0.05 versus Undifferentiated. Key: BSP, bone sialoprotein; OCN, osteocalcin; ACTA2, smooth muscle alpha 2 actin; TAGLN, transgelin. γOsteoblastic differentiation media details: Minimum essential medium eagle (Sigma-Aldrich, M8042) supplemented with 0.292 g/L L-glutamine (Sigma-Aldrich, G6392), 100 nM dexamethasone (Sigma-Aldrich, D4902), 50 μM ascorbic acid 2-phosphate (Sigma-Aldrich, 49752), 10 mM β-glycerophosphate (Sigma-Aldrich, G9422), 10% FBS (Sigma-Aldrich, F6178) and 1% Pen/Strep (Sigma-Aldrich, P4333). (TIF) [file pone.0188192.s005.tif]

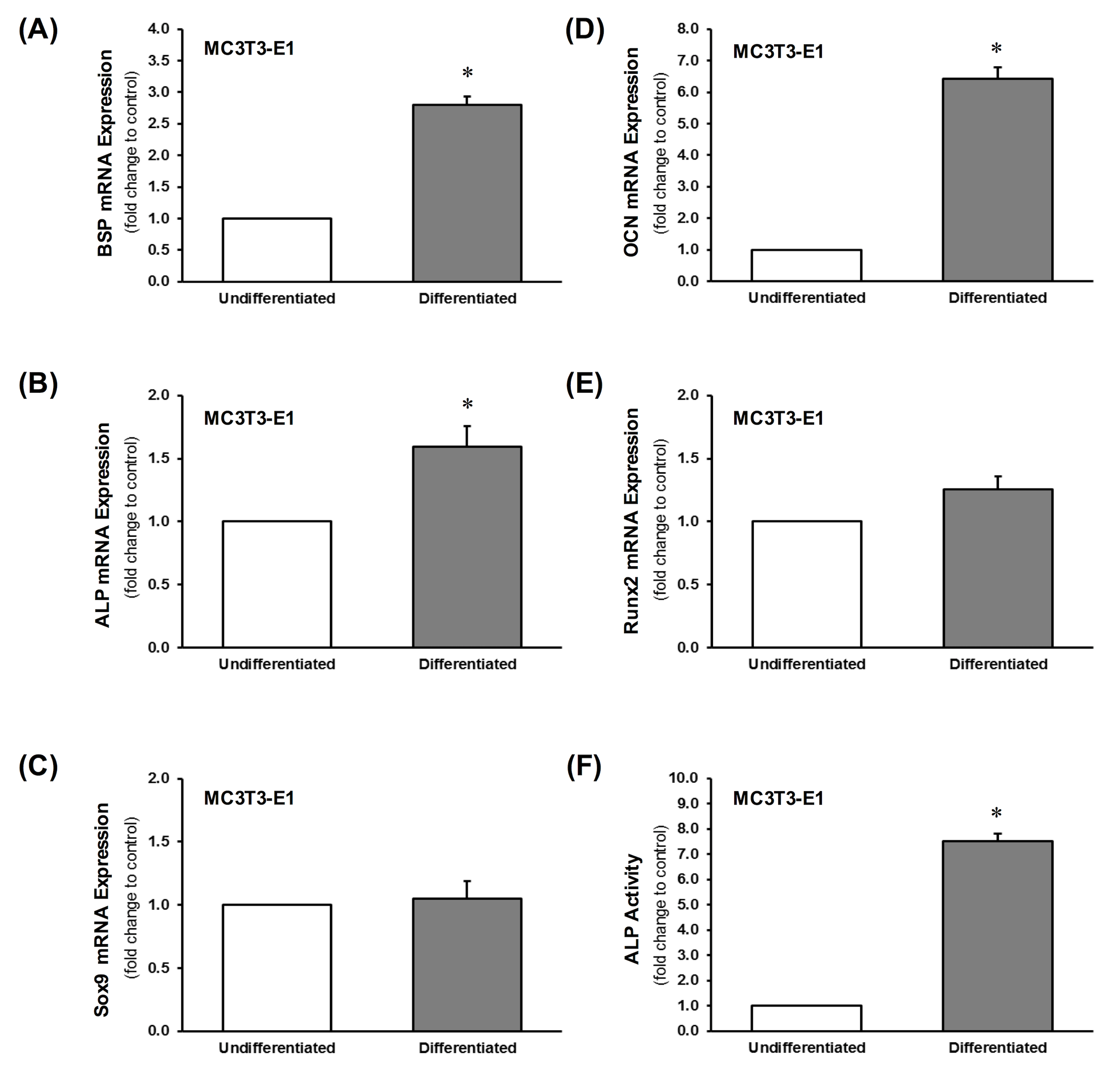

Supplement: S6 Fig — Cells were treated for 21 days with either standard media or osteoblastic differentiation media and then analyzed by qPCR for (A) BSP, (B) ALP, (C) Sox9, (D) OCN and (E) Runx2 mRNA. Conditioned media was also harvested and analyzed for (F) ALP enzymatic activity. *P≤0.05 versus Undifferentiated. γOsteoblastic differentiation media details: As outlined in S5 Fig above. (TIF) [file pone.0188192.s006.tif]

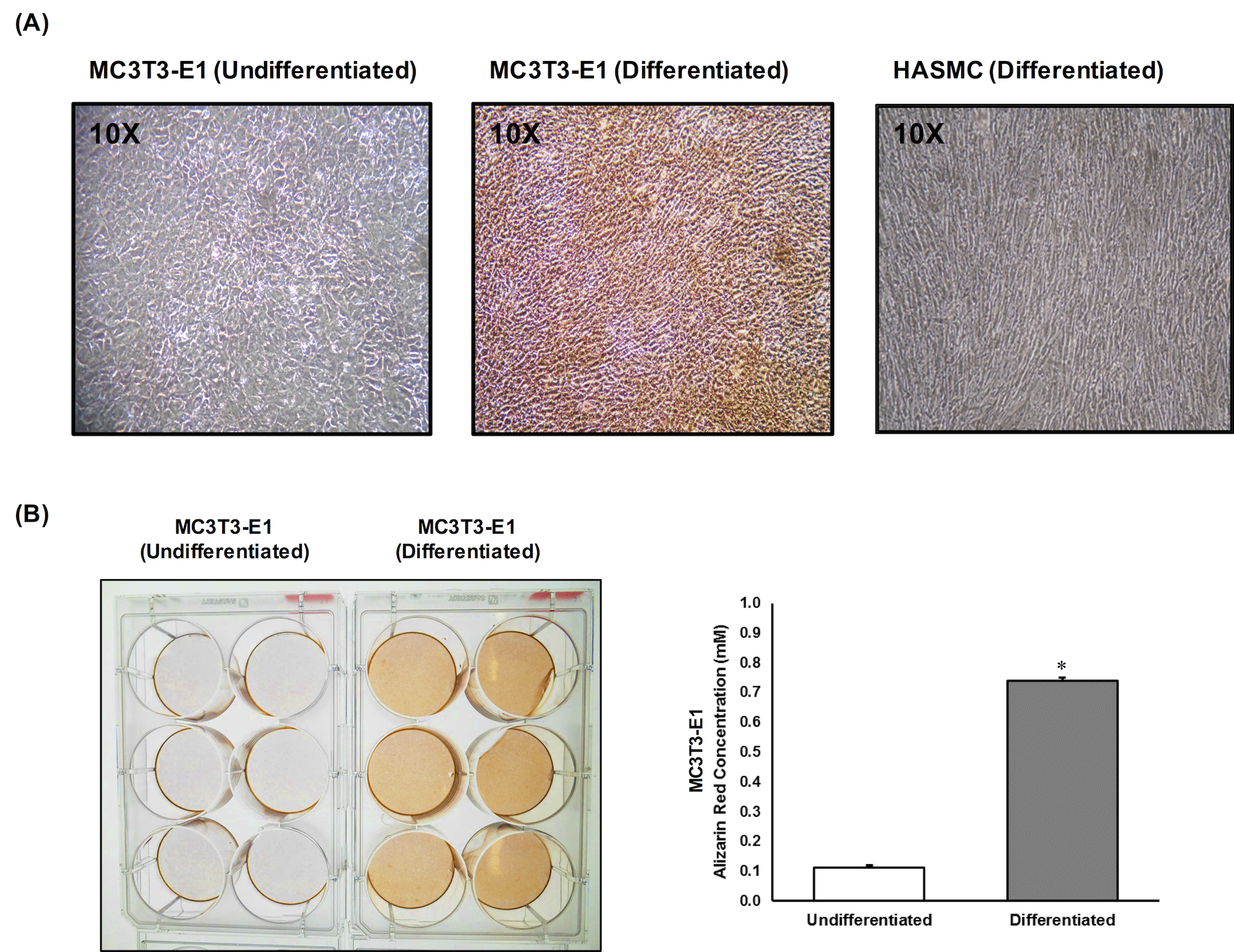

Supplement: S7 Fig — Cells were treated for 21 days with either standard media or osteoblastic differentiation media* and then analyzed by alizarin red staining for calcium deposition. Light microscopy showing alizarin red staining levels in MC3T3-E1 (but not in HASMCs) under 10X magnification (A) and for MC3T3-E1 cells in 6-well plates (B). Extraction of alizarin red dye from undifferentiated and differentiated MC3T3-E1 cells and quantitative spectroscopic analysis is also shown in the histogram (B, lower). *P≤0.05 versus Undifferentiated. γOsteoblastic differentiation media details: As outlined in S5 Fig above. (TIF) [file pone.0188192.s007.tif]
