# Supplementary material for: TRAIL attenuates RANKL-mediated osteoblastic signalling in vascular cell mono-culture and co-culture models
Source: PLoS One. 2017 Nov 16;12(11):e0188192. doi: 10.1371/journal.pone.0188192 (PMC5690591; doi:10.1371/journal.pone.0188192)
Supplement: S1 Supplementary Methods — (PDF) [file pone.0188192.s008.pdf]

Supplementary methods:

Culture of MC3T3-E1 pre-osteoblasts

MC3T3-E1 cells were cultured in either standard media (Minimum essential medium eagle containing 10% FBS and 1% Pen/Strep) or osteoblastic differentiation media (containing differentiation supplements as previously detailed in legend to S5 Fig), and were maintained in a humidified incubator at 37°C and 5% CO_2. Passages 22-26 were used for experimental purposes. Cells were trypsinized at approximately 90% confluency and cell viabilities were regularly checked using the ADAM™ counter. 21-day incubations began when cells were seeded into 6-well culture dishes, reaching confluency at approximately 5-7 days.

Alizarin Red staining: Microscopy

Spent media was removed and cells were washed three times with calcium- and magnesium-free PBS (Sigma-Aldrich, D8537). Cells were fixed with 3.7% formaldehyde (Sigma-Aldrich, F8775) for 15 minutes at room temperature, and washed a further three times with distilled H_2O. Alizarin Red staining solution (40mM, pH 4.1, sterile-filtered) (Merck Millipore, TMS-008-C) was added to the cells and incubated in the dark for 30 minutes with gentle agitation. The staining solution was then removed and cells washed three times with dH_2O. Cells were immediately imaged at 0X, 10X and 40X magnification.

Alizarin Red staining: Quantitation by absorbance

To extract the stain, water was removed and 10% (v/v) acetic acid was added to the wells. Cells were incubated for 30 minutes in the dark with gentle agitation. Cells were scraped, transferred to microcentrifuge tubes and vortexed for 30 seconds. Samples were then heated to

85ºC for 10 minutes and subsequently cooled on ice, before centrifugation at 20,000xg for 15 minutes. Supernatants were then neutralised with 10% ammonium hydroxide before loading in triplicate in an opaque-walled 96-well plate, which included an Alizarin Red standard curve (0-4 mM). Absorbance was measured at 405 nm.

Quantitative real-time PCR (qPCR)

Extraction of total RNA, preparation of cDNA and amplification of target cDNA sequences were carried out as previously described. Human GAPDH, ALP, Runx2 and Sox9 primer sequences were all found to be suitable for analysis of murine gene expression in MC3T3-E1 pre-osteoblasts. Additional primer sequences are as follows: Human BSP (79bp): Forward 5'-gaacctcgtggggacaattac-3'; Reverse 5'-catcatagccatcgtagccttg-3'. Human OCN (110bp): Forward 5'-ggcgctacctgtatcaatgg-3'; Reverse 5'-gtggtcagccaactcgtca-3'. Human ACTA2 (122bp): Forward 5'-ctatgagggctatgccttgcc-3'; Reverse 5'-gctcagcagtagtaacgaagga-3'. Human TAGLN (104bp): Forward 5'-ccgtggagatcccaactgg-3'; Reverse 5'-ccatctgaaggccaatgacat-3'. Murine BSP (148bp): Forward 5'-atggagacggcgatagttcc-3'; Reverse 5'-ctagctgttacacccgagagt-3'. Murine OCN (187bp): Forward 5'-ctgacctcacagatcccaagc-3'; Reverse 5'-tggtctgatagctcgtcacaag-3'.
